# Neuroprotective Properties of Rutin Hydrate against Scopolamine-Induced Deficits in BDNF/TrkB/ERK/CREB/Bcl2 Pathways

**DOI:** 10.3390/neurolint16050082

**Published:** 2024-09-27

**Authors:** Inturu Sreelatha, Ga-Young Choi, In-Seo Lee, Omkaram Inturu, Hyun-Sook Lee, Yea-Na Park, Cheol-Won Lee, Inkyou Yang, Sungho Maeng, Ji-Ho Park

**Affiliations:** 1 Department of Gerontology (AgeTech-Service Convergence Major), Graduate School of East-West Medical Science, Kyung Hee University, Yongin 17104, Republic of Korea; sree47@khu.ac.kr (I.S.); lis37@khu.ac.kr (I.-S.L.); jethrot@khu.ac.kr (S.M.); 2 Center for Research Equipment, Korea Basic Science Institute, Cheongju 28119, Republic of Korea; gayoung4613@kbsi.re.kr; 3 Department of Applied Physics and Institute of Natural Sciences, Kyung Hee University, Yongin 17104, Republic of Korea; omkar@khu.ac.kr; 4 Department of East-West Medicine, Graduate School of East-West Medical Science, Kyung Hee University, Yongin 17104, Republic of Korea; dongsimgi@naver.com (H.-S.L.); ta2627133@naver.com (Y.-N.P.); muinjidae@naver.com (I.Y.); 5 Convergence Healthcare Research Institute, Myong Ji University, Yongin 17058, Republic of Korea; 2846won@hanmail.net

**Keywords:** rutin hydrate, scopolamine, long-term potentiation, Alzheimer’s disease, synaptic plasticity

## Abstract

**Background/Objectives**: Alzheimer’s disease (AD) is an age-related degenerative brain disorder characterized by a progressive decline in cognitive function and memory. This study aimed to evaluate whether rutin hydrate (RH) has neuroprotective effects in an AD-like learning and memory impairment rat model induced by scopolamine (SCO). **Methods:** The rats were administered with RH (100 mg/kg) and SCO (1.5 mg/kg) and underwent behavioral tests, including the Morris water maze test, Y-maze test, and passive avoidance test, to evaluate their learning and memory abilities. Additionally, long-term potentiation (LTP) was induced to observe changes in the field excitatory postsynaptic potential (fEPSP) activity. **Results:** RH treatment attenuated the SCO-induced shortening of step-through latency in the passive avoidance (PA) test, increased the percentage of alternation in the Y-maze, and increased the time spent in the target zone in the Morris water maze (MWM). Moreover, RH increased the total activity of fEPSP following theta burst stimulation and attenuated the SCO-induced blockade of fEPSP. RH also ameliorated the SCO-induced decrease in the expression levels of the BDNF, TrkB, ERK, CREB, and Bcl-2 proteins and the increase in the Bax protein level in the rat hippocampus. This demonstrates that RH has beneficial neuroprotective effects in the brain, improving learning, memory, and synaptic plasticity in rats. **Conclusions:** Our results highlight the molecular and cellular mechanisms through which RH exerts its neuroprotective effects in the prevention and treatment of learning and memory deficit disorders. RH could potentially be used as a therapeutic strategy for the restoration of learning and memory function and the prevention of the progression of AD.

## 1. Introduction

Neurodegenerative diseases (NDD), such as Alzheimer’s (AD) and Parkinson’s (PD) diseases, are considered common age-related motor nerve disorders of chronic and fatal illnesses that establish a foremost danger to human health [1,2,3]. The proportion of the elderly population worldwide has significantly increased in size and signifies the fourth-highest basis of disease affliction in well-developed countries of high income. These diseases advance with age and remain incurable despite a massive amount of investment in healthcare and research [4]. The common indicator of neurodegenerative diseases [5], such as AD, is a cognitive impairment that results from the accumulation of amyloid beta (Aβ) plaques [6] and tau protein in deceased neural cells. Thus, NDD signifies some of the greatest challenges for elementary science and clinical medicine. A pharmacological report demonstrated the use of flavonoids in neurodegenerative conditions, especially AD and PD [7,8]. Considerable efforts in evaluating the chronic evolution characterized by the damage of memory demonstrated that herbal-derived polyphenol compounds with neuroprotective potential gained importance for NDD [5]. The characteristic polyphenolic structure is owing to the free radical foragers acting as antioxidants for the responsive oxygen species [9,10], which are mostly due to the varying effectiveness and efficacy of a single flavonoid to another, rendering the amount and location of OH groups prevalent to their structure, the grade of corrosion and unsaturation, as well as other replacements and conjugations related to their elementary method [11].

Rutin hydrate (RH), a well-known flavonoid, shows promising potential and health benefits owing to its strong anti-inflammatory and anti-hypertensive effects and potent antioxidant activity. RH reduces cognitive decline in an extensive range of NDDs by protecting against 6-hydroxydopamine-induced toxicity in rodents [12]. RH was used to analyze the phenolic components and antioxidant activity of nettle to evaluate the flavonoid content in propolis [13]. RH strengthens deregulation, reduces skin aging, and regulates enzymes in the extracellular matrix [14]. RH has been reported to attenuate colistin-induced oxidative stress, apoptosis, and inflammation [15]. The involvement of novel strategies by accepted bio-plants resulting in composites consumed as small supplements helps in the treatment of human diets with their natural food bases. Pharmacological studies demonstrated that RH and its derivatives defend dopaminergic neurons and alleviate apoptosis, astrogliosis, and oxidative stress 6-hydroxydopamine persuaded rodents of PD [16]. Autophagy activity has been studied in numerous models of Aβ-linked pathology to reverse Huntington’s disease by the insulin-like growth factor 1/insulin signaling pathway of RH. RH improves spatial memory and reduces the Aβ oligomer levels and neuroinflammation in a PS1/APP rat model of AD [17].

Scopolamine (SCO), a muscarinic receptor blocker, is a well-established tool for the pharmacological study of cognitive impairment in learning and memory in animals and humans. SCO administration causes cholinergic dysfunction, upregulation of tau proteins, a decrease in acetylcholine level, increased amyloid beta deposition, and induces cognitive disorders in Alzheimer’s disease [18]. Chronic SCO behavior also disrupts long-term memory retention in hippocampal brain-derived neurotrophic factors (BDNF) and cAMP response element-binding protein (CREB), which play significant roles in the regulation of the nervous system [19,20,21]. The neuronal transmission of long-term potentiation (LTP) involves postsynaptic function, and plasticity intensifies the regulation from a few hours to numerous weeks and, subsequently, the tedious electrical stimulus of synaptic nerves [22,23]. The N-methyl-D-aspartate receptor (NMDAR) generally induces LTP through the pathway for glutamate, which is covertly associated with hippocampal activity and facilitates learning and memory [24]. LTP has consequently been explored for assessing the function of cognitive synaptic mechanisms [25] by inducing cortical excitability changes using the rhythmically occurring theta burst stimulation (TBS) pattern, which improves the synaptic strength of neural networks.

This study primarily seeks to investigate the potential neuroprotective capacity of RH alone or in conjugation with SCO to induce neurotoxicity and cognitive impairment in rodents. RH seemed to improve spatial memory function by boosting the endogenous antioxidant pathway, activating cell viability, and improving apoptotic potential, which was more precise than when RH was conjugated with SCO. The neuroprotective effects of RH on learning and memory impairment induced by SCO were evaluated first by extensive electrophysiological assays using recently advanced microelectrode array (MEA) measurement methods. Second, we assessed and evaluated the hypothesis of short-term and long-term memory associated with well-established tests of rodents, such as the Y-maze task, passive avoidance (PA) test, and Morris water navigation task (MWN). Additionally, we also evaluated the association of neural-signaling-pathway-related protein levels in rodent hippocampal tissue to understand biological brain aging and reduce the risk of cognitive impairment, which was attributed to the positive effect of RH on NDD. Therefore, these results outline future opportunities and challenges associated with the use of RH as a promising neuroprotective compound for the treatment of NDD, such as AD.

## 2. Materials and Methods

### 2.1. Materials

Rutin hydrate (RH) and scopolamine (SCO) were purchased from Sigma-Aldrich (St. Louis, MO, USA). 4-(2-hydroxyethyl)-1-piperazineethanesulfonic acid (HEPES), dimethyl sulfoxide (DMSO), L-glutamine, Calcium Chloride (CaCl_2_), D-glucose, potassium chloride (KCl), magnesium chloride (MgCl_2_), sodium bicarbonate (NaHCO_3_), and sodium chloride (NaCl) were acquired from Sigma-Aldrich (St. Louis, MO, USA). Hank’s balanced salt solution (HBSS) and minimum essential medium (MEM) were purchased from the company Welgene Inc. (Gyeongsan, Gyeongsangbuk-do, Republic of Korea). The heat inactivated (HS) donor horse serum was obtained from Biowest in Nuaillé, France. Penicillin–streptomycin was obtained from Gibco BRL (Rockville, MD, USA). All the reagents were research-grade products.

### 2.2. Animals

Adult male Sprague-Dawley (SD) rats with an average body weight of 130 ± 10 g were purchased from Saeron Bio, Inc. (Uiwang, Kyunggi-do, Republic of Korea). A total of 48 male SD rats were randomly assigned to 4 groups (Control, SCO, RH, SCO + RH) for in vivo experiments, with each group comprising 12 rats. Among these 10 rats, 7 were allocated for behavioral tests, while the remaining 5 were designated for molecular analysis. All the rats were kept in cages in a 12/12-h (lights on/off 08:00 a.m./p.m.) light/dark cycle at a moisture level of 60 ± 5% and room temperature of 23 ± 1 °C in controlled atmosphere storage rooms. The individual cages housed two rats to ensure sufficient space among them; they were provided with tap water and a regular standard diet (Saeron Bio, Inc., Uiwang, Kyunggi-do, Republic of Korea) ad libitum during the course of the experiments. The experiments on the laboratory animals were carried out in a safe zone under the care guide of the committee of the Care and Use of Kyung-Hee University (KHGASP 19-337), which was approved by the supervisory principles of the Council of the National Institutes of Health Guide for the Care and Use of Laboratory Animals. Nearly all care was initiated to minimize the pain/distress experienced by all the laboratory animals to reduce the number of their use. The choice to use adult male Sprague-Dawley rats in this study was made to reduce variability associated with hormonal fluctuations, which could affect the interpretation of hippocampal function and neuroplasticity.

### 2.3. Experimental Procedure

**Figure 1:**
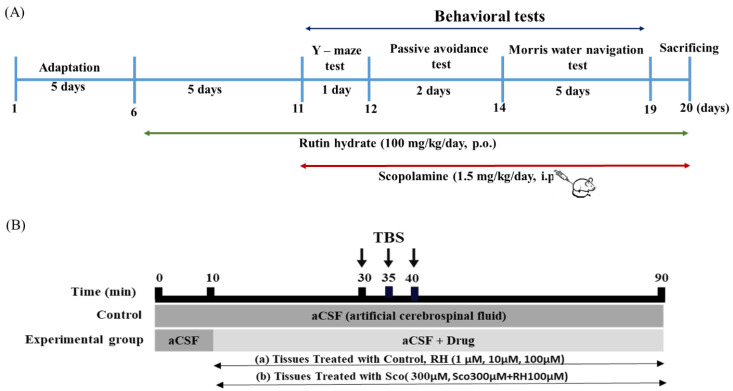
Experimental schedules for rutin hydrate effects. (**A**) Time schedule for behavioral tests. RH treatments were administered for a total of 15 days. Following pre-treatment, RH and SCO treatments were administered for 10 days for the behavioral tests. Y-maze test, passive avoidance test, and Morris water navigation test were performed for 8days. (**B**) Time schedule for electrophysiological tests. The organotypic hippocampal slices were treated with 300 µM SCO and RH (1, 10 100 µM) along with aCSF. Each experiment was executed for a total of 90 min and consisted of 30 min of base-line recording of fEPSP at one stimulation per min, 10 min of TBS, and 50 min of post-TBS fEPSP measurements. SCO, scopolamine; RH, rutin hydrate.

RH was administered per oral (p.o.) at a recommended dose of 100 mg/kg/day, p.o. body weight [26,27,28], and SCO was administered by intraperitoneal injection (i.p.) at 1.5 mg/kg/day, i.p. body weight of the recommended dose [29]. The rats were arbitrarily divided into four groups (Table 1). The rats were adapted for 5 days, and then RH treatments were administered for a total of 15 days, and SCO treatments were administered for 10 days for the behavioral tests. RH was administered p.o. for 60 min, and SCO i.p. was injected for 30 min before each test. For 8 consecutive days, the responses of behavioral testing for all the pretreatment were evaluated with 7 rats of each group. The rats were sacrificed on the last day (Figure 1).

### 2.4. Hippocampal Slice of Organotypic Cultures (OHSCs)

The hippocampal slices of organotypic cultures (OHSCs) were arranged with interfaces defined in the studies [30] using seven-day-old male SD (Sprague-Dawley) rats procured from Saerone Bio Inc (Uiwang, Kyunggi-do, Republic of Korea). The brain was dissected quickly by immediately immersing it in a cold HBSS medium with 20 mM of HEPES. Furthermore, the hippocampal slices were enucleated and sectioned into 350 μm-thick slices by using a tissue chopper (Mickle Laboratory Engineering Co. Ltd, Gomshall, Guildford, UK) to rapidly make acute cerebellar slices from rats. Six slices were plated on top of 0.4 µm membrane of insert (CM-Millicell; Millipore, Bedford, MA, USA) in a 6-well cultured plate and maintained at 35 °C with 5% CO_2_ in an incubator. The six-well plate was filled with 1 mL of MEM—50%(*v*/*v*) culture medium (heat-inactivated horse serum; 25%(*v*/*v*), HBSS; 25%(*v*/*v*), supplemented with 20 mM HEPES, 5.25 g/L D-glucose, 1 mM L-glutamine, and 1% penicillin–streptomycin, pH = 7.1). On every second day, the culture medium was changed, and the hippocampal slices were grown for days (12–14) in an incubator kept at 35 °C and then used for the experiments.

### 2.5. Preparation of Organotypic Hippocampal Slice Tissue on Microelectrode Array (MEA)

The solitary stabilized hippocampal portion tissue remained prudently isolated after a membrane of the inset through a brush and was positioned on a MEA 8 × 8 of ten (10) μM thickness electrodes, which remained pre-coated with polyethyleneimine of 0.01% (Multi-Channel Systems, Reutlingen, Baden-Württemberg, Germany). The multi-channel systems contain an amplifier with an array of high-density electrodes (MEA1060), stimulator (STG1004), computer software (MC_Rack), and controlled temperature unit. All the sliced tissues were steadied in artificial cerebrospinal fluid (aCSF, pH 7.4) containing 114 mM NaCl, 25 mM NaHCO_3_, 25 mM glucose, 3 mM KCl, 2 mM CaCl_2_, 1.3 mM MgCl_2_, 20 mM HEPES, and 1.1 mM NaH_2_PO_4_ for 30 min with 95% O_2_ and 5% CO_2_ gas aeration at 33 °C. The hippocampus tissue slice was transferred to an amplifier interface (MEA1060) and stabilized perfectly with the help of an Ag/AgCl pellet. The channel of stimulation was excluded after recording the stimulation process [31].

### 2.6. Induction of Long-Term Potential (LTP) in Hippocampal Slices

The point of inducing the Schaffer collateral and commissural pathway was chosen based on the morphological examination of hippocampal tissue and an appropriate response to bipolar electrical stimulation. As a result, it was applied to the hippocampus CA 3 region and was placed in a Faraday cage. The intensity of bipolar stimulation was set at 160 μA and applied to 240 ms per phase, and the optimum level was required to produce 40–65% of the maximum hippocampal tissue reaction. The theta burst stimulation (TBS) consisted of 300 biphasic pulses administered in three trains of 100 Hz for 1 sec with 5 min intervals [32]. Each experiment of LTP induction was performed for a total of 90 min, consisting of 30 min of a field excitatory postsynaptic potential (fEPSP) record, 10 min of TBS, and 50 min of post-TBS fEPSP measurement once per min. During experiments, the slices were continuously infused with fresh aCSF bubbled with 95% O_2_ and 5% CO_2_ at a rate of 3 mL/min and were treated with Control, RH (1, 10, 100 μM), SCO (300 μM), and SCO (300 μM) + RH (100 μM) from 10 min after the start of recording. All unfiltered data were sampled from 60 recording channels at 25 kHz using the recording system of MC_Rack (v.3.2.1.0, Multi-Channel Systems, Reutlingen, Baden-Württemberg, Germany).

### 2.7. Y-Maze Task

The Y maze task is valuable in assessing the short-term working memory in rodents. An apparatus consisted of a Y-shaped maze of black polypropylene walls with three opaque arms spread 120° apart (10 × 45 × 35 cm). The arms were labeled as “A”, “B”, and “C”, and the rat was placed at the intersection of three arms and allowed to move freely for 10 min without the presence of stimuli such as water or food. Entry into an arm was recorded if all four paws of the rats were inside the arm. Spontaneous alternation performance is defined as visiting three different arms consequently in ABC, ACB, BCA, and BAC orders. Alternative arm return is defined as visiting other arms and re-turning to the same arm, such as ABA, ACA, BAB, BCB, CAC, and CBC orders. When the rat entered the three arms consecutively, spontaneous alternation was recorded, and the following formula was used to calculate the percentage of spontaneous alternation [33]: [(number of spontaneous alternations)/(total number of arm entries − 2)] × 100.

### 2.8. PA Test

The PA test is a distress intensified test that is used to assess the effect of chemical entities on learning and memory and study the mechanism and substantial influence on the cognitive processes in rodents [34]. The PA shuttle box chamber consisted of equal-sized light and dark compartments (17 cm × 12 cm × 15 cm) separated by a black guillotine gate (9 cm × 17 cm), which was kept open to allow passage. The floor was designed using steel gratings with a 2.5 mm diameter and connected to an electric shock stimulator, which transports electricity to the grid floor in the dark compartment. The experiment comprised two different trials: a training session for the acquisition of fear and a retention session for detecting whether the memory of fear remained. During the training session, a rat was placed in the light compartment and left for 60 s after the middle gate was opened until the rat passed into the dark compartment. The following day, when four feet of the rat were inside the dark compartment, the door was suddenly closed, and a mild electric shock (1.5 mA, 50 Hz, 1.5 s) was delivered. The rat was then removed from the dark chamber and returned to the cage for 20 s. The retention and fear memory periods were performed 24 h and 72 h after the training sessions. Rodents learn to associate certain properties of the chamber with the foot shock; they are subsequently placed back to test their learning and memory and placed back in the compartments where no shock was delivered. Rodents learn to avoid electrical shocks by entering the adjacent compartment using normal learning and memory [35]. During a retention session, a rat was once again positioned in the light chamber for 60 s, and the step-through latency time to enter the dark chamber was measured up to a cut-off time of 600 s.

### 2.9. MWN Task

The MWN task evaluates long-term spatial learning and memory using distal cues to navigate from the starting locations around the perimeter of an open water tank to locate a submerged escape platform for finding and measuring its ability [36]. A circular water tank (Diameter—180 cm × Height—45 cm) was filled with pure water (25 ± 1 °C) to a depth of 35 cm and rendered opaque by adding non-toxic white paints. A plat-form for escape was positioned randomly on one of the four quarters divided into an equal size of the tank and kept hidden by a 1 cm submersion underneath the surface of the water. Four visual arts with different shapes were randomly assigned to the north–south–east–west quadrants of the tank. All the rats were trained for four days in different quadrants at each session. Visible platform training followed for four days, starting at four different regions of the water tank each day. During the training period, the latency required to reach the underwater platform was recorded. If the rat failed to find the platform within 60 s, the rat was allowed to rest on the platform for 20 s. If the rat failed to locate the platform within 60 s, the experimenter guided the platform passively and allowed them to remain on the platform for 20 s. During the test period, navigation was carried out for the rats that were allowed to navigate for 90 s without a platform. The total experimental work was recorded by an SHC-650A video camera (Samsung, Suwon, Republic of Korea) located directly above the water tank. Moreover, the results of detecting a navigation path, latency to trace the platform, remaining time, and extra parameters were assumed using the SMART v30 video tracking software of Panlab Harvard Apparatus, Barcelona, Spain.

### 2.10. Western Blot

Dissected brain tissues from rats in the Normal Control, SCO, RH, SCO + RH groups were analyzed by Western blotting. Subsequent to the behavioral tests, the hippocampus was homogenized in the pre-cooled RIPA buffer to prepare a complete all-in-one cocktail containing both phosphatase and a complete protease inhibitor. The homogenized tissues were incubated on ice with a shaker for 60min, and the supernatant was obtained by centrifuging the solution at 14,000 rpm, 4 °С for 20 min. The measured protein concentrations, at a wavelength of 562 nm of supernatants, were quantified by a Bradford protein assay [37], and equivalent quantities (10 μg) of protein remained partitioned on an electrophoresis gel with ten percent SDS-PAGE, the divided protein gels were directly transferred onto PVDF membranes. All the membranes were incubated in 5% skim milk for 60 min and then treated with main antibody overnight at 4 °C with agitation. Rabbit anti-BDNF (ab108319, Abcam, Cambridge, UK), monoclonal antibody against extracellular signal-regulated kinase (ERK, ab184699), phospho-ERK (ab201015), CREB (ab32515), phospho-CREB (ab254107), Tropomyosin receptor kinase B (TrkB, ab18987), phospho-TrkB (ab229908), B-cell lymphoma 2 (Bcl2, ab59348), and Bcl2 associated X protein (Bax, 2772S, Cell Signaling Technology Inc., Danvers MA, USA)were used for the experiments. After incubating with the first antibody, the membrane was incubated for 60min at room temperature with 5% skim milk containing a secondary anti-body such as goat anti-rabbit (7074S, Cell Signaling Technology Inc., MA, USA), IgG conjugated to horseradish. After a final wash step, the membrane was incubated with an appropriate enzyme substrate to generate a recordable signal with EzWestLumi plus ECL solution (WSE-7120S, ATTO Co., Tokyo, Japan) using a system for the task of detecting instances with Ez-Capture II (ATTO Co., Tokyo, Japan). All experiments were repeated in triplicate and above with different biopsy-sized tissue samples, for their findings are more reproducible.

### 2.11. Electrophysiology Data Processing

All of the data were sampled from 60 recording channels at 25 kHz using the Recorder-Rack software. The data, consisting of analog MEA signals with MC_Rack (v.3.2.1.0, Multi-Channel Systems, Reutlingen, Germany), were converted to digital form, of which EPSPs over 80 mV were selected using the trigger mode. To stimulate and integrate the evoked field potential trajectory, a custom MATLAB program (v.7.0.1, Mathworks, Inc., Natick, MA, USA) was used to eliminate stimulus artifacts [38,39].

### 2.12. Statistical Analysis

The statistical analysis was performed using a well-known software SPSS (version 25.0, IBM SPSS Statistics Inc., IL, USA), and significant changes in the mean values were investigated with the help of a one-way analysis of variance (ANOVA) with least significant difference (LSD) post hoc test (*p* < 0.05). All of the data are expressed as mean ± standard error of the mean (SEM).

## 3. Results

### 3.1. RH Enhances Fepsp Activity of LTP in the CA1 Region of Hippocampal Slices

**Figure 2:**
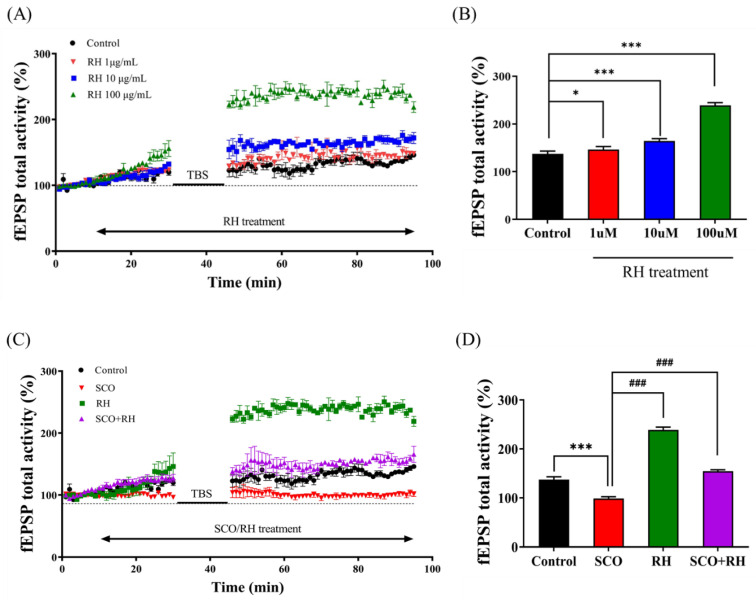
Effect of RH on LTP in the rat hippocampus (n = 5) group. (**A**) Time course of total fEPSP activity percentage variation induced by TBS during the treatment of RH in the organotypic-cultured hippocampus. (**B**) Average of fEPSP total activity 30 to 40 min post-TBS during the dose-dependent treatment of RH (1, 10, and 100 μM). Value of 1 μM (* *p* < 0.05), value of 10 μM (*** *p* < 0.001), value of 100 μM (*** *p* < 0.001) vs. a control group. (**C**) Time course of total fEPSP activity percentage change induced by TBS during the treatment of SCO vs. SCO + RH in the organotypic cultured hippocampus. (**D**) Average of fEPSP total activity 30–40 min following TBS during the treatment of SCO and SCO + RH. *** *p* < 0.001 vs. control group, ^###^ *p* < 0.001 vs. SCO group. Significant values between the pairs of groups examined by one-way ANOVA in SPSS analysis of LSD variance statistically significant difference test. SCO, scopolamine; RH, rutin hydrate.

The primary objective was to investigate the dose dependence of RH treatment (1, 10, and 100 μM) on LTP of the CA1 hippocampal region and assess the hypothesis that RH enhances synaptic plasticity. The time-interval percentage change in fEPSP activity (Figure 2A) and change in the activity of fEPSP and the percentage of the mean from 30 to 40 min post-TBS (Figure 2B) were calculated for data analysis [F(3, 12) = 277.237, *p* < 0.001]. The total fEPSP activities of the 1 μM RH group (*p* < 0.05) were slightly different from those of the control group. The average post-TBS activity of fEPSP between 30 and 40 min demonstrated that the 1 μM RH was higher than that of the control group (*p* < 0.05). In the 10 μM RH group, the total activities of fEPSP and average post-TBS activity of fEPSP from 30 to 40 min were both increased and associated with the control group (*p* < 0.001). Furthermore, in comparison with the control group, the total activities of fEPSP and average post-TBS time of 30–40 min fEPSP activity for the 100 μM RH group were increased (*p* < 0.001).

### 3.2. RH Alleviates Impairment of LTP Induction by SCO in the CA1 Region of Hippocampal Slices

Second, the impact of RH on SCO-induced LTP impairment was examined in the SCO and SCO + RH groups. Tissue from each group was treated with 300 μM SCO and 300 μM SCO + 100 μM RH. The total activity of fEPSP in the SCO group was favorably lower than that of the control (Figure 2C). In contrast, the SCO + RH group demonstrated increased SCO levels. The average post-TBS activity of the fEPSP assessment from 30 to 40 min in each group was different [F(3, 16) = 433.605 *p* < 0.001]. The SCO group demonstrated reduction compared with the experimental group of control average post-TBS activity of fEPSP (*p* < 0.001). The average post-TBS fEPSP activity rate of the SCO + RH (*p* < 0.001) group improved compared to the SCO group (*p* < 0.001), which was near to the value of the control group. These results indicate that RH improved LTP dominance induced by SCO (Figure 2D).

### 3.3. RH Amends Spatial Learning Short-Term Memory Deficits in the Y-Maze Task 

**Figure 3:**
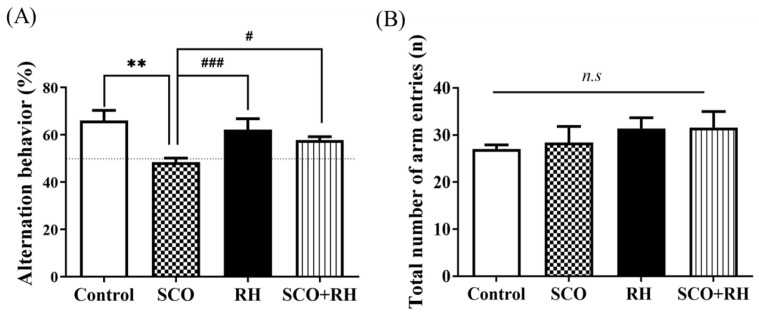
(**A**) The properties of RH on SCO-induced spatial memory effect in Y-maze (n = 5). The proportion of spontaneous alternations in Y-maze test was calculated. Data are presented as mean ± SEM and ** *p* < 0.01 vs. control group, ^#^ *p* < 0.05 and ^###^ *p* < 0.001 vs. SCO group. (**B**) Total number of maze arm entries into 3 arms. No significant differences were observed between all the groups. Final data are expressed as mean ± SEM. Note that non-significant (*n.s*) differences were observed in any of the parameters among the groups. Significant values between the pairs of groups examined by one-way ANOVA in SPSS analysis of LSD variance statically significant difference test. SCO, scopolamine; RH, rutin hydrate.

The Y-maze task determined the effect of RH on spatial memory and learning impairment in SD rat models; the alternation behavior test was performed using the Y-maze. The results indicated that the ratio of spontaneous alternation in each group was significantly different [F (3, 16) = 7.348, *p* < 0.01; Figure 3A], The SCO group demonstrated decreased behavioral alterations compared with the control group (*p* < 0.01). The percent of alteration in the Y-maze was significantly above chance level (50%) in the control but not in the SCO group. Meanwhile, the RH (*p* < 0.001) and SCO + RH groups significantly increased (*p* < 0.05) compared with the SCO group. The RH and SCO + RH groups alternated at levels that were above chance (50%). There was no change in the total number of maze arm entries [F (3, 16) = 0.710, *p* < 0.560, (Figure 3B)], which indicates that the differences in behavioral alternation had no spectacular influence on locomotor activity for spatial memory.

### 3.4. RH Enhances Long-Term Spatial Learning and Memory Deficits in the MWN Task

**Figure 4:**
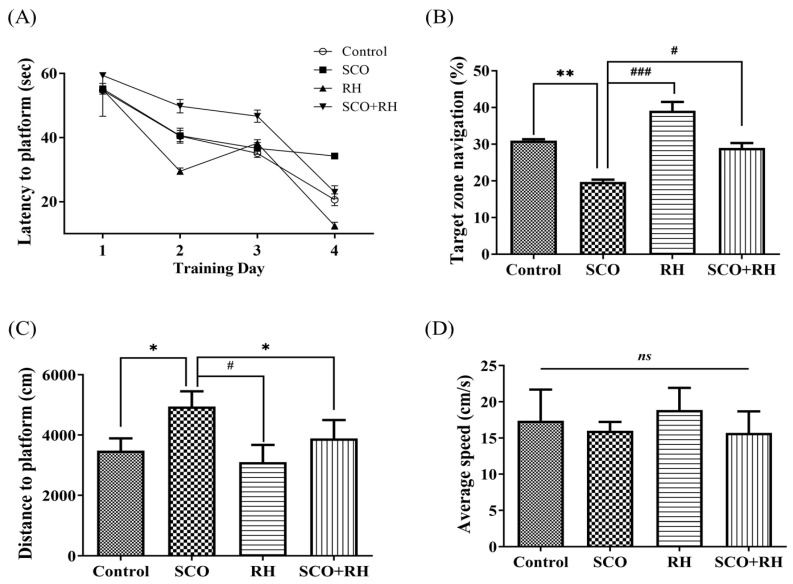
The result of RH on long term spatial learning and memory in the MWN task. (**A**) Escape latency to the concealed platform during 4 days of training. (**B**) Percentage of time spent in the target zone during the test session. (**C**) Mean distance to platform in the test session. (**D**) Average speed during the test session. All the data are presented as mean ± SEM, n = 4, * *p* < 0.05, ** *p* < 0.01 vs. control, ^#^ *p* < 0.05, ^###^ *p* < 0.001 vs. SCO. Note that non-significant (*ns*) differences were observed. The values are examined by one-way ANOVA in SPSS with post hoc LSD test. SCO, scopolamine; RH, rutin hydrate.

The MWN task was used to assess the effects of RH on SCO-induced long-standing spatial learning and memory deficits in an SD rat model by MWN pool experiment. The rats were trained in the MWN task for four trials per day for four consecutive days (Figure 4A). The escape latency demonstrated deterioration over the training days in all the groups [F(3,16) = 3.526, *p* < 0.05]. In the SCO group, the latency was markedly longer than that in the control group on all the training days (*p* < 0.05). After a 4-day training session, a test was conducted to check the time spent in the located platform quadrant [F(3, 16) = 15.806, *p* < 0.001, Figure 4B]. Rodents in the control (*p* < 0.01) group and RH/SCO groups spent more time in the target quadrant, while SCO treatment significantly reduced (*p* < 0.01) time in the target quadrant. In the RH (*p* < 0.001) group and SCO + RH co-treated rats, the target zone time demonstrated a significant (*p* < 0.05) increase in the locomotor activity of the SCO group. In the comparison of distance to the platform, the SCO group showed an increase compared to the control group (*p* < 0.05). However, the SCO + RH group exhibited a reduction in comparison to the SCO group (*p* < 0.05, Figure 4C). There was no discernible difference in average speed across the groups (Figure 4D). The results of the MWN test demonstrated that learning and memory capabilities could be impaired by SCO; however, RH ameliorated this impairment.

### 3.5. RH Ameliorates Fear Avoidance Learning and Memory Deficits in the PA Test

**Figure 5:**
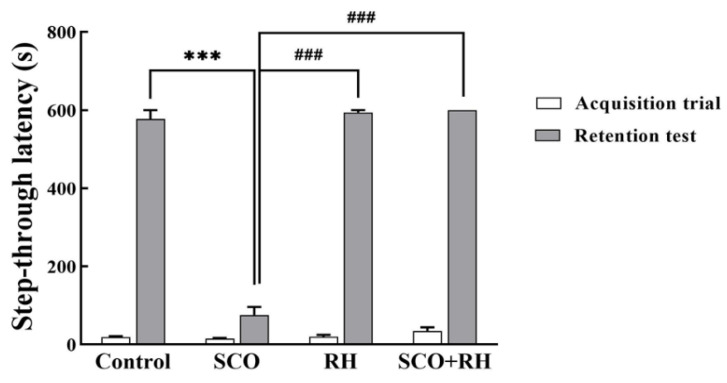
The results of RH on the step through latencies on the SCO-induced SD rats in the trial session of the avoidance task; the rats were trained to adapt to the step-through avoidance apparatus. When the rats entered the dark room compartment, they received an electrical shock. The 24 h retention session was conducted after the training session. The following considerations examined the latency to entering the dark room compartment. Final data are presented with n = 5, mean ± SEM, *** *p* < 0.001 vs. control, ^###^ *p* < 0.001 vs. SCO group, followed by the post hoc statistically significant difference test analyzed by LSD one-way ANOVA. SCO, scopolamine; RH, rutin hydrate.

The PA test examined the effect of RH on SCO-induced learning and memory deficits in SD rat models, the fear-avoidance memory test was performed as shown in Figure 5. The rats received a trial session for the acquisition of fear and a retention session of the PA test following the Y-maze task. In the trial session, no significant differences were observed in the latency to enter the dark room of the passive avoidance apparatus compartment between all the groups [F (3, 16) = 0.827, *p* = 0.504]; no significant differences were observed between most of the outcome groups with electrical shock. In the 24 h retention session, which was tested following the trial session [F(3, 16) = 267.055, *p* <0.001], the SCO treatment group show a significant reduction compared to the control group in the step-through latency of the retention test (*p* < 0.001). The RH group (*p* < 0.001) and SCO + RH group demonstrated significant increase compared with the SCO group (*p* < 0.001). These findings revealed that SCO interrupts passive avoidance memory preservation and RH improves the retrieval of SCO-induced insults.

### 3.6. RH Upregulates BDNF, Tropomyosin Receptor Kinase B (TrkB), Extracellular Signal-Regulated Kinase (ERK), and CREB Expression in the Hippocampus of SCO-Induced SD Rat Model

**Figure 6:**
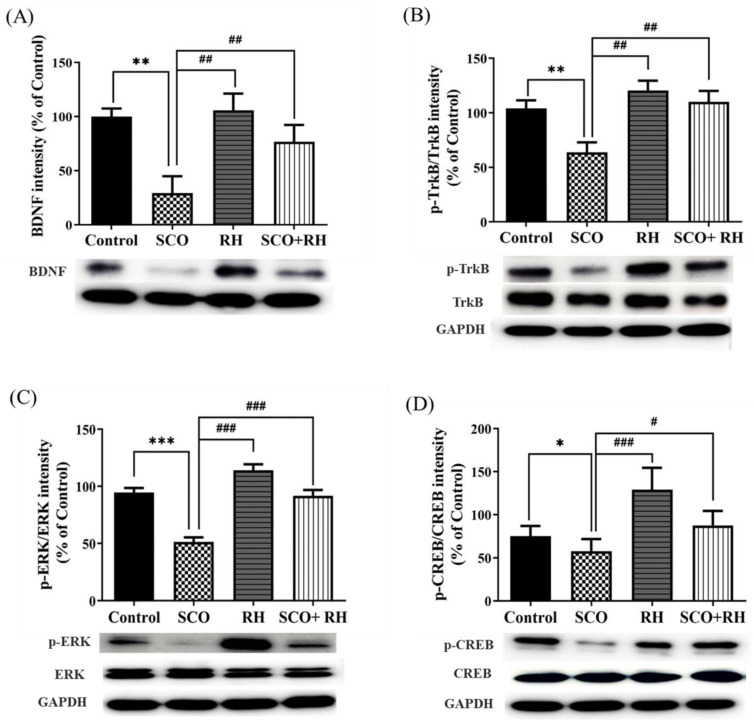
Effect of RH on the BDNF, TrkB, ERK, and CREB protein expression in the SCO-induced rat hippocampus. (**A**) Band quantification of BDNF/GAPDH ratios. (**B**) Band quantification of p-TrkB/TrkB/GAPDH ratios, (**C**) Band quantification of p-ERK/ERK/GAPDH ratios, (**D**) Band quantification of p-CREB/CREB/GAPDH ratios. Final data are presented when n = 5, mean ± SEM, * *p* < 0.05, ** *p* < 0.01, *** *p* < 0.001 vs. control, ^#^ *p* < 0.05, ^##^ *p* < 0.01, ^###^ *p* < 0.001 vs. SCO group with the analysis performed using one-way ANOVA honest significant difference LSD test between groups. SCO, scopolamine; RH, rutin hydrate.

Western blotting was performed to determine the effect of RH on the hippocampal BDNF-TrkB-CREB pathway (Figure 6). Considering the expression levels of BDNF [F (3, 16) = 5.402, *p* < 0.01, Figure 6A], the SCO group demonstrated a significant decrease compared to the control group (*p* < 0.01). Meanwhile, RH co-treatment caused an increase compared to that in the SCO group (*p* < 0.05). The p-TrkB/TrkB expression level in the SCO group (*p* < 0.01) signaling shows a downregulating compared to the control group [F(3, 16) = 5.008, *p* < 0.01, Figure 6B]. Additionally, the RH group and SCO + RH group increased in the signal level of TrkB in comparison to the SCO group (*p* < 0.01). The expression level of p-ERK/ERK in the SCO group was significantly downregulated compared to the control group (*p* < 0.001), as indicated by the signaling [F(3, 16) = 32.1, *p* < 0.001, Figure 6C]. In addition, both the RH group and the SCO + RH group exhibited a higher signal level of p-ERK/ERK compared to the SCO group (*p* < 0.001). In order to find the p-CREB/CREB expression [F(3, 16) = 14.6, *p* < 0.01, Figure 6D], the range of the SCO group is remarkably reduced related to the control group (*p* < 0.05). However, when SCO + RH was treated, the protein level of p-CREB/CREB increased significantly relative to the SCO group (*p* < 0.05). These results support the possibility that RH treatment would improve the activation of the BDNF-TrkB-ERK-CREB pathway in the hippocampus.

### 3.7. RH Regulates Apoptosis-Related Protein Production in the Hippocampus of SCO-Induced SD Rats

**Figure 7:**
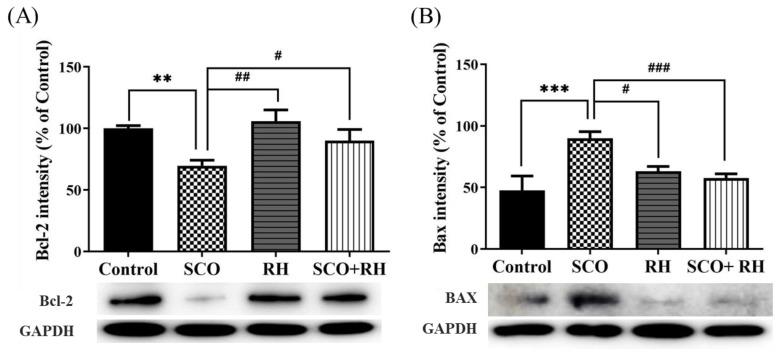
Results of RH on the protein expression of Bcl2 and Bax in SCO-treated rat hippocampus. Western blotting biomarkers of Bcl2, Bax, and GAPDH expression in the hippocampus (**A**) Band quantification of Bcl2/GAPDH ratios. (**B**) Band quantification of Bax/GAPDH ratios. Data are shown when n = 5, mean ± SEM, ** *p* < 0.01, *** *p* < 0.001 vs. control, ^#^ *p* < 0.05, ^##^ *p* < 0.01, ^###^ *p* < 0.001 vs. SCO group with the analysis performed using one-way ANOVA with post hoc LSD test between groups. SCO, scopolamine; RH, rutin hydrate.

Protein levels related to neuronal apoptosis in the hippocampus of SCO-induced SD rats were measured using Western blot analysis. The results of all the groups were moderate compared with those of the control group. The ratios of Bax/glyceraldehyde 3-phosphate dehydrogenase (GAPDH) and Bcl2/GAPDH showed significant differences in all the groups compared to the SCO group. For the apoptosis inhibitory protein Bcl2, RH was used, and a significant difference was observed among the handling groups [F (3, 16) = 5.267, *p* < 0.05, Figure 7A]. In association with the control group, Bcl2 levels were reduced in the SCO group (*p* < 0.01). Nevertheless, Bcl2 levels in the RH treated group (*p* < 0.05) were notably higher than those in the SCO group. An obvious change was observed in the hippocampal Bax appearance among the groups [F(3, 16) = 6.796, *p* < 0.01, Figure 7B]. The apoptosis-promoting protein Bax was significantly upregulated in the SCO group actively compared to the control group (*p* < 0.01), whereas the Bax levels in the RH (*p* < 0.05) and SCO + RH (*p* < 0.05) treatment group decreased in comparison with the SCO group. These results indicated that RH treatment could prevent SCO-induced apoptotic changes.

## 4. Discussion

In an aging society, NDD results in the slow progressive loss of neurons in the central nervous system (CNS), which leads to deficits in specific brain functions. Studies on the natural plant bioactive polyphenolic compound, RH, have been shown to inhibit cognitive impairment in scopolamine-induced SD rats. SCO is known as a non-selective muscarinic receptor antagonist that inhibits muscarinic acetylcholine receptors and central cholinergic neuronal activity. It causes deficits in learning and short-term memory and impairs cognitive and non-cognitive functions. Therefore, scopolamine has been used as an effective standard or reference component for the induction of dementia-related cognitive deficits, as well as the suppression of working memory (spatial) in healthy humans and animals [40,41,42]. Furthermore, the results of the previous studies showed that SCO injection (a daily dosage of 1.5 mg/kg/day, i.p.) successively blocked ACh receptors and induced cognitive dysfunction [43,44,45]. SCO administration (1.5 mg/kg/day, i.p.) in rats significantly elevated the neuroinflammatory markers, leading to neuronal injury [29,46]. Therefore, the concentration of SCO (1.5 mg/kg/day) was determined based on the previous studies.

In our study, the effect of RH on LTP induction was investigated using electrophysiology. Primary LTP experiments were performed to determine the optimal dose concentrations of RH (1, 10, and 100 μM), and the fEPSP total activity baseline percentage was measured between 30 and 40 min following TBS. All the groups treated with RH concentrations demonstrated significant differences from the control group and increased fEPSP total activity (Figure 7A,B). The results with the greatest difference were found at 100 μM, and the results confirmed that this optimal concentration increased the fEPSP total activity. LTP is a key experimental model for measuring synaptic activity related to learning and memory [47,48,49,50]. LTP can also be used to study NDD, such as AD, and the factors that cause neuroinflammation, which can result in AD [51]. The results demonstrated that RH potentiated CA1 LTP and ameliorated SCO-induced impairment of LTP (Figure 2C,D). RH treatment enhanced LTP in ex vivo cultured hippocampal tissue [52] cultures at doses of 1, 10, and 100 µM compared to the control group, and RH of 100 µM increased the spontaneous activity of fEPSP prior to TBS. Thus, 100 µM RH was used to assess the effects of RH on SCO-treated tissues. Notably, treatment with 100 µM RH attenuated SCO-induced LTP impairment in tissues. The total fEPSP activity in the scopolamine group was favorably lower than that of the control, whereas the SCO + RH group increased with the SCO group (Figure 2C,D). Thus, RH enhances synaptic strength and has a protective effect against SCO-induced neurodegeneration [53]. Learning and memory require synaptic plasticity arbitrated by NMDA and AMPA glutamate receptors that comprise LTP, which rapidly increases the synaptic strength [54]. In response to acute stimulation and homeostatic plasticity, which gradually adjust the synaptic strength in response to chronic activity variations, LTP and homeostatic plasticity are expressed through variations in AMPA receptor localization and function. Alterations in these mechanisms are implicated in nervous system disorders, including AD, schizophrenia, Down syndrome, and autism. Moreover, localized Ca^2+^ influx [55] through NMDAR can provide emerging CA3 pyramidal cell synapses and may be involved in activity-dependent synaptogenesis and network formation. Activation of NMDAR leads to a cytosolic free intracellular calcium (Ca^2+^) increase, which is required for LTP and synaptic plasticity [56]. Cognitive impairment in AD is closely related to synaptic plasticity, in which NDMAR plays a critical role. Excitatory glutamatergic neurotransmission via NDMAR is critical for synaptic plasticity and neuronal survival. Extracellular Aβ and tau work together to drive healthy neurons into a diseased state in the brain in AD, leading to aberrant excitatory network activity and compensatory inhibitory responses involving learning and memory circuits [57].

In the Y-maze test, the measured percentage of spontaneous alternation was connected to short-term spatial memory, and rats preferred exploring a new arm rather than returning to the previously visited arm, which is consistent with rodents’ urge to explore new habitats. The findings showed that the proportion of spontaneous alternations varied considerably across groups (Figure 3A). The RH group exhibited a substantial difference, whereas the SCO group showed less alternation behavior than the control group. There were no differences in the total number of arm entries (Figure 3B), demonstrating that the variations in alternation behavior had no confusing effect on locomotor activity for spatial memory.

The MWN task is a generally acknowledged approach for evaluating long-term spatial learning and memory in rats. In the experimental trials, the escape latency to discover the platform revealed how rapidly the rats could learn and recall the location of the hidden platform, hence, evaluating long-term spatial learning ability. In this study, the escape latency to the platform of all the groups reduced progressively over four days [58]. However, the SCO group exhibited a slower learning rate than the others (Figure 4A). After training to assess the spatial learning ability of rats, the swimming time in the target zone where the platform was placed was assessed. The SCO group moved randomly and irregularly in the maze, and the swimming time of the rats across the target zone reduced. The performance deterioration of the SCO group suggested significant long-term learning and memory impairment in rats induced by SCO. When compared to the control, the RH+SCO group performed much more accurate navigation to the target quadrant and comparable swimming durations in the target zone (Figure 4B). These results suggest that RH may successfully attenuate long-term spatial learning and memory deficits.

The PA task is an experiment that uses the natural preference of the rat for darkness [59]. The PA test was performed to evaluate short-term learning and memory abilities in rats that were exposed to an inescapable electrical shock upon entering a dark box in this study. The SCO group did not remember about the electric shock of the previous day and entered the dark compartment again with little latency [60]. However, the RH group considerably slowed the step-through latency. RH administration substantially mitigated the reduction of the step-through latency caused by SCO. These findings suggest that RH inhibited short-term learning and memory deficits (Figure 5). In all the behavioral tests (Y-maze, PA, and MWN), RH effectively reduced learning and memory dysfunction induced by SCO.

Brain-derived neurotrophic factor (BDNF) is an important member of the classic neurotrophin family that plays a crucial role in the formation, development, and function of the brain via the BDNF-TrkB-CREB pathway, which regulates neuronal survival, differentiation, and plasticity by activation. BDNF, TrkB, and CREB are well-known indicators of synaptic function regulation, neuronal survival, and differentiation. These proteins may also play pivotal roles in hippocampal LTP and synaptic plasticity enhancement [61]. In this study, SCO treatment decreased the BDNF, TrkB, and CREB levels in rat hippocampi. These results are consistent with the previous research [62,63,64]. RH reduces acetylcholinesterase activity and inhibits it in certain brain regions, while increasing choline acetyltransferase, thereby elevating acetylcholine levels. Based on this, RH regulates BDNF/TrkB signaling as an acetylcholine agonist [65]. The ERK/MAPK pathway links extracellular signals to membrane receptors, triggering a cascade reaction in transcription factors and eventually regulating gene expression. This pathway has been confirmed to be involved in neurodegeneration and oxidative stress [66]. RH increased the expression of these three proteins, thereby attenuating SCO-induced downregulation in the hippocampus. Bcl2 family proteins are central regulators of mitochondria-mediated apoptosis and are involved in neural apoptosis [67]. Bcl2 enhances cell survival, and Bax promotes cell death [68]. Bcl2 has also been suggested to be directly dependent on the elevation of Bax. Moreover, a few reports have demonstrated that Bax protein levels increase in the brain of AD patients [69,70]. Considering the effect of RH on Bcl2 and Bax, the results of the Western blot analysis demonstrated that RH attenuated decreasing and increasing Bax and Bcl2 expression, respectively. RH was shown to suppress neuronal histone acetylase activity and increase histone-tail acetylation, which enhanced the expression of BDNF-TrkB signaling pathway components, including CREB, independent from the activation of nuclear factor erythroid 2-related factor 2 (Nrf2), suggesting that RH could enhance neuronal plasticity. Furthermore, clinical studies have investigated the safety profile and efficacy of flavonoids against peptic ulcers, schizophrenia, autism spectrum disorder, and other diseases [71,72,73]. Despite the many advantages of RH, such as its DMSO/dimethyl formamide (DMF) solubility, good pharmacokinetics, safety during oral administration, and potential blood–brain barrier penetration ability [74], doses of 150–300 mg/kg have been reported to produce marked sedation, hyperthermia, impaired motor coordination, and decreased skeletal muscle length in mice [75]. Therefore, the safety profile and risk-to-benefit ratio of RH usage should be comprehensively evaluated.

## 5. Conclusions

In conclusion, our evaluated results demonstrated that RH prevented the suppression of LTP and enhanced short- and long-term spatial recognition and avoidance memory in scopolamine-induced SD rats. The protective effects of RH were associated with upregulation of the expression of proteins related to the BDNF/TrkB/ERK/CREB pathway and the reduction of proapoptotic activity via Bcl2 and BAX. These findings indicate that RH improves learning and memory abilities by triggering cell survival and inhibiting apoptosis pathways. In addition, the electrophysiological analysis demonstrates that RH enhances synaptic plasticity and synaptic function in the hippocampus through LTP. Consequently, RH may be a worthwhile agent for the treatment of diseases of learning and memory impairment.

## Figures and Tables

**Table 1:** Animal grouping and experimental schedule.

Groups	Subjects	Treatment
Group 1	Control	PBS 1 mL
Group 2	SCO treated	SCO 1.5 mg/kg/day, i.p.
Group 3	RH	RH 100 mg/kg/day, p.o
Group 4	SCO + RH	SCO 1.5 mg/kg/day, i.p. + RH 100 mg/kg/day, p.o.

## Data Availability

The data used to support the findings of this study are available from the corresponding author upon request.
